# Nucleofection-based screening of chimeric antigen receptor candidates in human natural killer cells

**DOI:** 10.3389/fimmu.2025.1557766

**Published:** 2025-04-03

**Authors:** Chelsia Qiuxia Wang, Leonard Leong, Lei Yang, Shengli Xu, Kong-Peng Lam, Andre Boon-Hwa Choo, Andy Hee-Meng Tan

**Affiliations:** ^1^ Immune Cell Manufacturing, Bioprocessing Technology Institute (BTI), Agency for Science, Technology and Research (ASTAR), Singapore, Singapore; ^2^ Biotherapeutics Development, Bioprocessing Technology Institute (BTI), Agency for Science, Technology and Research (ASTAR), Singapore, Singapore; ^3^ Singapore Immunology Network, Agency for Science, Technology and Research (ASTAR), 8A Biomedical Grove, Immunos, Singapore, Singapore; ^4^ Departments of Physiology, Yong Loo Lin School of Medicine, National University of Singapore (NUS), Singapore, Singapore; ^5^ Departments of Microbiology and Immunology, Yong Loo Lin School of Medicine, National University of Singapore (NUS), Singapore, Singapore; ^6^ Departments of Pediatrics, Yong Loo Lin School of Medicine, National University of Singapore (NUS), Singapore, Singapore; ^7^ School of Biological Sciences, Nanyang Technological University (NTU), Singapore, Singapore; ^8^ Department of Biomedical Engineering, Faculty of Engineering, National University of Singapore (NUS), Singapore, Singapore; ^9^ Food, Chemical and Biotechnology Cluster, Singapore Institute of Technology (SIT), Singapore, Singapore

**Keywords:** chimeric antigen receptor (CAR), natural killer (NK) cells, nucleofection, mRNA, screening

## Abstract

Chimeric antigen receptor (CAR)-modified cell therapy products approved for clinical treatment of hematological malignancies have hitherto been based on T cells. NK cells represent a promising immune cell type that can be considered for CAR engineering due to their potential to be generated as off-the-shelf allogeneic cellular therapy. Viral transduction of NK cells with CARs has been fraught with challenges of long process time and poor CAR transduction efficiency. Here, we describe the development of an optimized protocol for electroporation-based delivery of CAR mRNA into NK cells expanded from human peripheral blood mononuclear cells in the presence of co-stimulating feeder cells. This enabled rapid assessment of the functional capacity of NK cells transiently expressing various CARs to kill liquid and solid tumor cells *in vitro*. Ultimately, we anticipate that such an approach will enable selection of CAR candidates for their subsequent clinical applicability and manufacturability.

## Introduction

Natural killer (NK) cells, being part of the innate immune system, are a highly promising cell type for tumor immunotherapy due to their potential to be generated as an off-the-shelf allogeneic product (1, 2). NK cells can target infected or transformed host cells directly via release of perforin and granzymes and also indirectly through secretion of various cytokines and chemokines that recruit adaptive immune cells (3). Their HLA-independent intrinsic killing capability and alloreactivity against malignant cells are conferred by an array of endogenous activating receptors such as NKG2D and inhibitory receptors such as killer-cell immunoglobulin-like receptors (KIRs) (3). To date, clinical trials have demonstrated that infusing NK cells into tumor patients are safe as they presented with almost no incidence of graft-versus-host disease (GvHD), cytokine release syndrome and neurotoxicity (4–7). Although the endogenous activating receptors can serve to elicit its innate cytotoxic effects, introduction of CARs into NK cells can enhance their antigen-specific anti-tumor cytotoxicity and persistence. At present, six CAR-T cell therapy products have been clinically approved and prescribed for autologous use, while the safety and potency of CAR-NK cells are still being evaluated in multiple clinical trials (8).

Typically, CAR transgenes are packaged into lentiviruses or retroviruses which are transduced into NK cells, resulting in stable and long lasting CAR expression in the cells (9). However, this method is time consuming and results in low and variable frequency of CAR-modified NK cells, which reduces its effectiveness in screening for CARs that enhance the anti-tumor function of NK cells. CAR DNA plasmids or CRISPR-Cas9 ribonucleoprotein complexes (RNPs) encompassing CAR DNA templates have also been introduced into NK cells via non-viral transfection (10, 11). In particular, electroporation of primary T cells with DNA plasmids have been shown to adversely affect cell viability (12), while similar delivery of CRISPR-Cas9 RNPs incurs substantially higher cost and likelihood of undesirable off-target genome editing.

In this study, we developed a protocol that would enable rapid, comparative evaluation of CARs to mediate tumor-killing efficacies in NK cells. We report an optimized process in which mature NK cells from peripheral blood are expanded using feeder cells and subsequently subjected to nucleofection with *in vitro* transcribed CAR mRNAs in place of DNAs. This not only generated substantially high frequency of CAR^+^ NK cells but also alleviated death typically observed after electroporation of NK cells. CAR expression, albeit transient, lasted for sufficient duration in NK cells to allow high-performing CARs to be robustly distinguished from low-performing candidate CARs in a high throughput manner.

## Materials and methods

### Cell culture

Suspension tumor cell lines (HL-60, MOLM14, U937, MV4;11 and K562) were cultured in complete RPMI medium (cRPMI, Nacalai Tesque #30264-56) supplemented with 10% fetal bovine serum (FBS, Hyclone SV30160.03). PC-9 cells were cultured in cRPMI medium (Gibco #11875-093) supplemented with 10% FBS and 2mM L-glutamine (Gibco #25030-081). SKOV3 cells were cultured in DMEM (high glucose, Gibco, #11960-044) and DMEM (low glucose, Gibco, #11885-084) at a 1:1 ratio, and supplemented with 10% FBS. All cells were maintained in humidified 37°C incubator with 5% CO_2_.

### Feeder cell-based NK cell expansion

NK cells were expanded from human peripheral blood mononuclear cells (PBMCs, STEMCELL Technologies) as described previously (13). Briefly, cells were thawed in a 37°C water bath and washed twice in pre-warmed complete Biotarget™ medium (cBiotarget, Biological Industries, Sartorius #05-080-1A) supplemented with 10% FBS (Hyclone SV30160.03), 100 unit/ml penicillin, 100 μg/ml of streptomycin (Sartorius #03-031-1B) and 4 mM L-glutamine (Gibco #25030-081). Cell viability and density were determined using 0.2% w/v Trypan blue solution (Sigma #T6146) in phosphate-buffered saline (PBS). Retrieved PBMCs were then co-cultured with 100-Gy irradiated K562 feeder cells engineered to express membrane-bound (mb) IL-15, mbIL-21 and 4-1BB ligand (13) at a ratio of 1:2 in cBiotarget medium supplemented with 10 IU/ml human IL-2 (Peprotech #200-02-1000 or #200-02-500). Cell cultures were maintained every two days by replenishing 50% of the cBiotarget medium supplemented with 10 IU/ml IL-2. On day 7 or day 8, CD3^+^ T cells were depleted from the culture using CD3 microbeads (Miltenyi Biotech #130-050-101) and LS column (Miltenyi Biotech #130-042-401) following manufacturer’s instructions. The remaining CD3^-^ NK cells were cultured at a density of 2 × 10^6^ cells/ml in cBiotarget medium supplemented with 50 IU/ml IL-2 and 10 ng/ml IL-15 (STEMCELL Technologies #78031.2) until the day of electroporation.

### Activation of T cells

PBMCs (STEMCELL Technologies) were similarly thawed as described above in cRPMI media (Gibco #11875-093) and activated in the presence of bead-bound anti-CD3 and anti-CD28 antibodies (Gibco #11132D), 20 U/ml recombinant human IL-7 (Miltenyi Biotec #130-095-362), 10 U/ml recombinant human IL-15 (Miltenyi Biotec #130-095-764) and 0.04 U/ml recombinant human IL-21 (Miltenyi Biotec #130-095-769) for 3 days. Electroporation was carried out on day 3.

### Flow cytometry

Prior to antibody (Ab) staining, all cells were treated with Human TruStain FcX (Fc receptor blocking solution; BioLegend #422302). Surface markers of expanded NK cells were analyzed by staining with LAG3-FITC (#11-2239-42) and CD96-PE (#12-0969-42) from eBiosciences, CD3-APC-Cy7 (#300426), CD16-PerCP-Cy5.5 (#302028), CD226/DNAM-1-FITC (#338303), TIGIT-PE-Cy7 (#372713), NKG2D-BV711 (#563688), TIM3-PE-Cy7 (#345013) and CD57-PerCP-Cy5.5 (#359621) from Biolegend, CD56-BV510 (#563041), NKG2A-BV510 (#747922), NKG2C-BV650 (#748165) and PD-1-PerCP-Cy5.5 (#561273) from BD Biosciences, and KLRG1-FITC (#130-103-705) from Miltenyi Biotec. CAR expression in nucleofected NK cells was either reported by the percentage (%) of eGFP^+^ as surrogate marker for CAR or detected by staining with recombinant biotinylated protein L (ACROBiosystems #RPL-P814R) followed by PE-conjugated streptavidin (eBioscience #12-4317-87). 4’,6-diamidino-2-phenylindole dihydrochloride (DAPI; Biolegend #422801) or Hoechst 33342 (Invitrogen #H3570) solution were used to exclude dead cells. Samples (at least 2 × 10^4^ events) were acquired on MACSQuant X (Miltenyi Biotech) or BD LSR II (BD Biosciences) to assess expression. Data was analyzed with FlowJo software (TreeStar).

### *In vitro* transcription (IVT) of CAR constructs

The different CAR constructs were cloned into pcDNA3.1(+) backbone vector (GenScript). 2 μg DNA template was linearized by overnight restriction enzyme digestion with XbaI. The linearized template was then purified using phenol/chloroform method, and reconstituted in 5 μl of water. The purified template was used for *in vitro* transcription using the HiScribe T7 ARCA mRNA kit (with tailing) (NEB #E2060S), following the manufacturer’s protocol. The mRNA quality was assessed by running denatured RNA samples on a 1% agarose gel containing 0.5% bleach at 80 V for 90 min. The mRNA concentration was measured by Nanodrop and the respective CAR mRNA molar concentration was calculated. Single-use aliquots of 66 nmol were stored at -80°C before use.

### Generation of CAR-NK cells

To generate CAR-NK cells following expansion, IVT CAR mRNA was introduced into NK cells via electroporation using P3 Primary cell 4D-Nucleofactor X Kit (Lonza #V4XP-3032 or #V4XP-3024) and program CM-137 in 4D-Nucleofector X Unit (Lonza). Electroporated cells were then expanded in cBiotarget media in the presence of 500 IU/ml IL-2 and 20 ng/ml IL-15 for 24 h. Where T cells were used as positive control, the nucleofection program EO-115 was used and cells were returned to cRPMI media in the presence of 20 U/ml recombinant human IL-7, 10 U/ml recombinant human IL-15 and 0.04 U/ml recombinant human IL-21.

### *In vitro* cytotoxicity assay using luciferase-based method

NK cells were co-cultured with a fixed number (1.125 × 10^4^) of luciferase-expressing HL-60 cells at effector cell: target cell (E:T) ratios ranging from 1:1 to 10:1 in 96-well plate for 16-20 h. Surviving tumor cells were assessed for associated luciferase activity employing the Bright-Glo Luciferase Assay System (Promega #E2620) conducted essentially according to the manufacturer’s protocol. 75 μl of culture medium was mixed with 75 μl of the prepared luciferase reagent in each well and the plates were shaken for 5 min to allow complete lysis of cells. Luminescence of the lysed mixture was measured using the Synergy HTX Multi-Mode Microplate Reader (BioTek). Percentage (%) cytotoxicity was calculated as: Δluc [luc (no CAR-NK) - luc (CAR-NK)]/luc (no CAR-NK) × 100%, where luc represents absolute luciferase units.

### *In vitro* cytotoxicity assay using xCELLigence platform

For experiments using adherent target cells, PC-9 and SKOV3, tumor cell growth was monitored in real-time using the xCELLigence platform (Agilent). 5 × 10^3^ target cells in 100 μl were seeded into each well in an E-plate 96 (Agilent # 5232376001) and co-cultured with NK cells at E:T ratios ranging from 1:1 to 10:1 at 37˚C.

### Statistical analyses

Differences in numerical values between samples used in *in vitro* cytotoxicity assays were compared by multiple unpaired student’s t-test (for parametric data sets with 2 groups) or by two-way ANOVA with the Tukey *post hoc* analysis (for parametric data sets with 4 groups) using Prism GraphPad Software (version 8). In all tests, a value of *p* < 0.05 for a given comparison was regarded as statistically significant.

## Results

### NK cells were expanded from cryopreserved PBMCs

**Figure 1:**
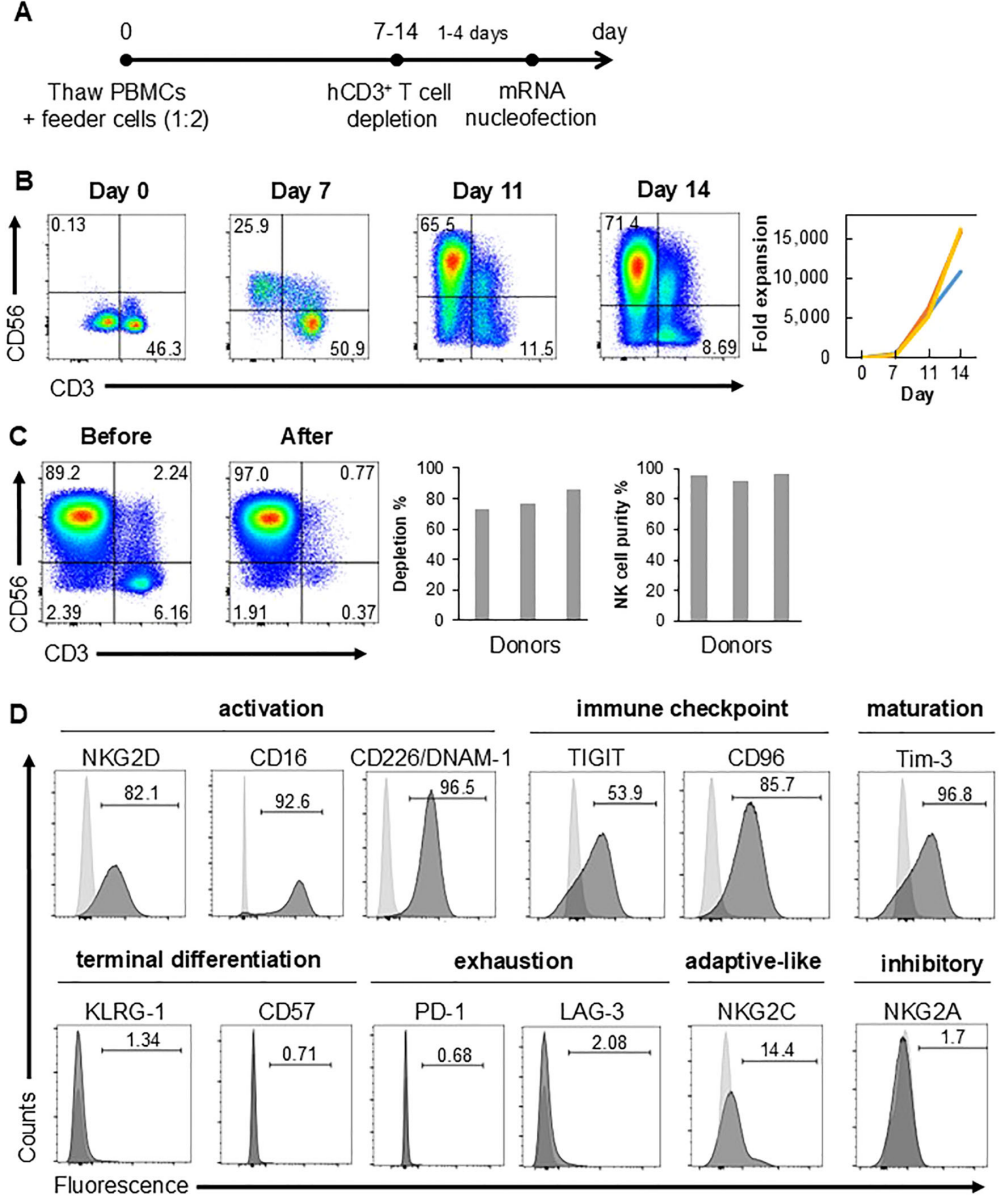
NK cells are expanded from cryopreserved PBMCs. **(A)** Schematic diagram showing timeline of NK cell expansion and CD3^+^ T cell depletion. **(B)** Proportions of CD3^-^ CD56^+^ NK and CD3^+^ CD56^-^ T cells gated on DAPI^-^ viable cells at various culture time points as assessed by flow cytometry (left). Fold expansion of CD3^-^ CD56^+^ NK cells at indicated time points relative to day 0 with each graph representing a different donor (right). **(C)** Proportions of CD3^-^ CD56^+^ NK cells gated on DAPI^-^ viable cells before and after CD3^+^ T cell depletion (left) as assessed by flow cytometry. Percentage (%) of CD3^+^ cell depletion, calculated as: [(total CD3^+^ cells before depletion – total CD3^+^ cells after depletion)/total CD3^+^ cell before depletion] x 100%, and % purity of CD3^-^ CD56^+^ NK cells (right). **(D)** % of cells expressing various cell surface molecules in DAPI^-^ viable cells after CD3^+^ cell depletion as assessed by flow cytometry. Light gray histograms depict fluorescence minus one controls which were used for gating. Data shown are representative of at least three independent healthy donors.

NK cells from cryopreserved PBMCs were expanded using a feeder-based system, employing irradiated K562 cells which have been engineered to express membrane-bound (mb) IL-15, mbIL-21 and 4-1BB ligand (13). PBMCs were co-cultured with feeder cells at 1:2 ratio on day 0 (**Figure 1A**). Over a period of 14 days, CD56^+^ CD3^-^ NK cells expanded at least 10,000-fold in the culture (**Figure 1B**). In contrast, feeder-free expansion protocol generated approximately 2,000-fold expansion after 28 days (10). CD3^+^ T cells which is the other major cell type were depleted from the culture at least one day before mRNA nucleofection by magnetic separation using CD3 microbeads. Flow cytometry analysis confirmed at least 70% depletion efficiency and >90% CD56^hi/+^ CD3^-^ NK cell purity after depletion (**Figure 1C**). A majority of the remaining NK cells expressed NK cell activation markers (NKG2D, CD16 and CD226/DNAM-1), immune checkpoint molecules (TIGIT, CD96), maturation marker Tim-3, but not terminal differentiation markers (KLRG-1 and CD57) nor exhaustion markers (PD-1 and LAG-3) (**Figure 1D**). Of note, about 15% of expanded NK cells were positive for another NK cell activation maker, NKG2C, which marks the adaptive-like NK cells during infection with human cytomegalovirus (14). Expression of NK cell inhibitory receptor NKG2A was negligible (**Figure 1D**). These data clearly reinforce the efficacy of feeder cell-based NK cell expansion from peripheral blood.

### An optimized nucleofection condition was required for the introduction of CAR mRNA into NK cells

**Figure 2:**
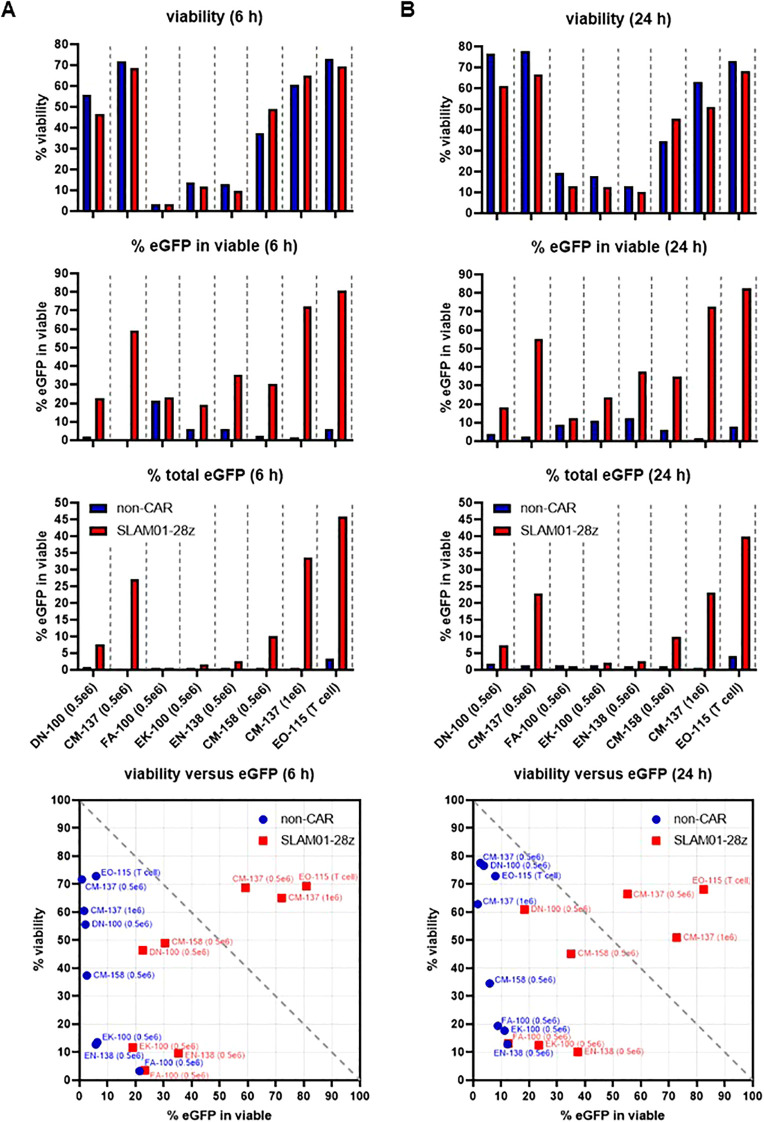
CM-137 is the optimal program for nucleofection of CAR mRNA into NK cells. **(A, B)** NK cells were electroporated using various Lonza nucleofector programs. % viability of (top row panels) and % eGFP within viable (second row panel) and total (third row panel) cells were assessed at 6 h **(A)** and 24 h **(B)** post-electroporation. Data were summarized in scatter plots to identify the most optimal nucleofection conditions concurrently maximizing viability of and eGFP expression in NK cells (bottom panels). Data shown are representative of 2 independent experiments.

As there was no precedence from previous literature reporting Lonza nucleofection conditions optimized specifically to electroporate mRNA into NK cells, we decided to test several conditions defined by varying nucleofection programs and cell densities. For these experiments, we used an in-house designed CAR construct, SLAM01-28z-IRES-eGFP, that carries an eGFP surrogate marker. We carried out two rounds of optimization, showing results representative of the latest round (**Figure 2**, **Supplementary Figure S1**). In each experiment, T cells were also electroporated with its respective optimized program, EO-115, to serve as positive control (**Figures 2A, B**, last column of each panel and **Supplementary Figure S1B**). Three programs, FA-100, EK-100 and EN-138 resulted in extremely poor viability of NK cells at both 6 h and 24 h post-electroporation (**Figures 2A, B**, top panel, columns 3-5). NK cells electroporated with three other programs, DN-100, CM-137 and CM-158, yielded higher viability, of which CM-137 achieved the highest, yielding more than 60% viable cells at both 6 h and 24 h time points (**Figures 2A, B**, top panel, columns 1, 2 and 6). Moreover, CM-137 was superior in producing the highest frequency of eGFP^+^ within viable and consequently, total cells (**Figures 2A, B**, second and third panels, column 2). In summary, visualization of our data via scatter plots correlating % viability with % eGFP expression identified optimal nucleofection conditions concurrently maximizing viability of and eGFP expression in NK cells, revealing the superior performance of CM-137 program compared with others we tested (**Figures 2A, B**, bottom panels).

Because we found CM-137 condition to yield high eGFP expression in and yet preserve viability of NK cells during the first round of experimental optimization (data not shown), we tested two different cell densities, namely 0.5 × 10^6^ and 1 × 10^6^ cells, on the effect of CM-137 in separate 20 μl nucleocuvettes. There were no significant differences with either cell density, albeit % eGFP expression slightly increased in cells seeded at higher density (**Figures 2A, B, first**, second and third panels, compare columns 2 and 7). Therefore, we conclude that the optimized condition for mRNA nucleofection into human NK cells is CM-137 program with 1 × 10^6^ cells per 20 μl nucleocuvette (or correspondingly, 5 × 10^6^ cells per 100 μl nucleocuvette) followed by a 24-hour resting period before subsequent assays were carried out.

### Optimized nucleofection condition is validated using 2448-28z and My96-28z CARs

**Figure 3:**
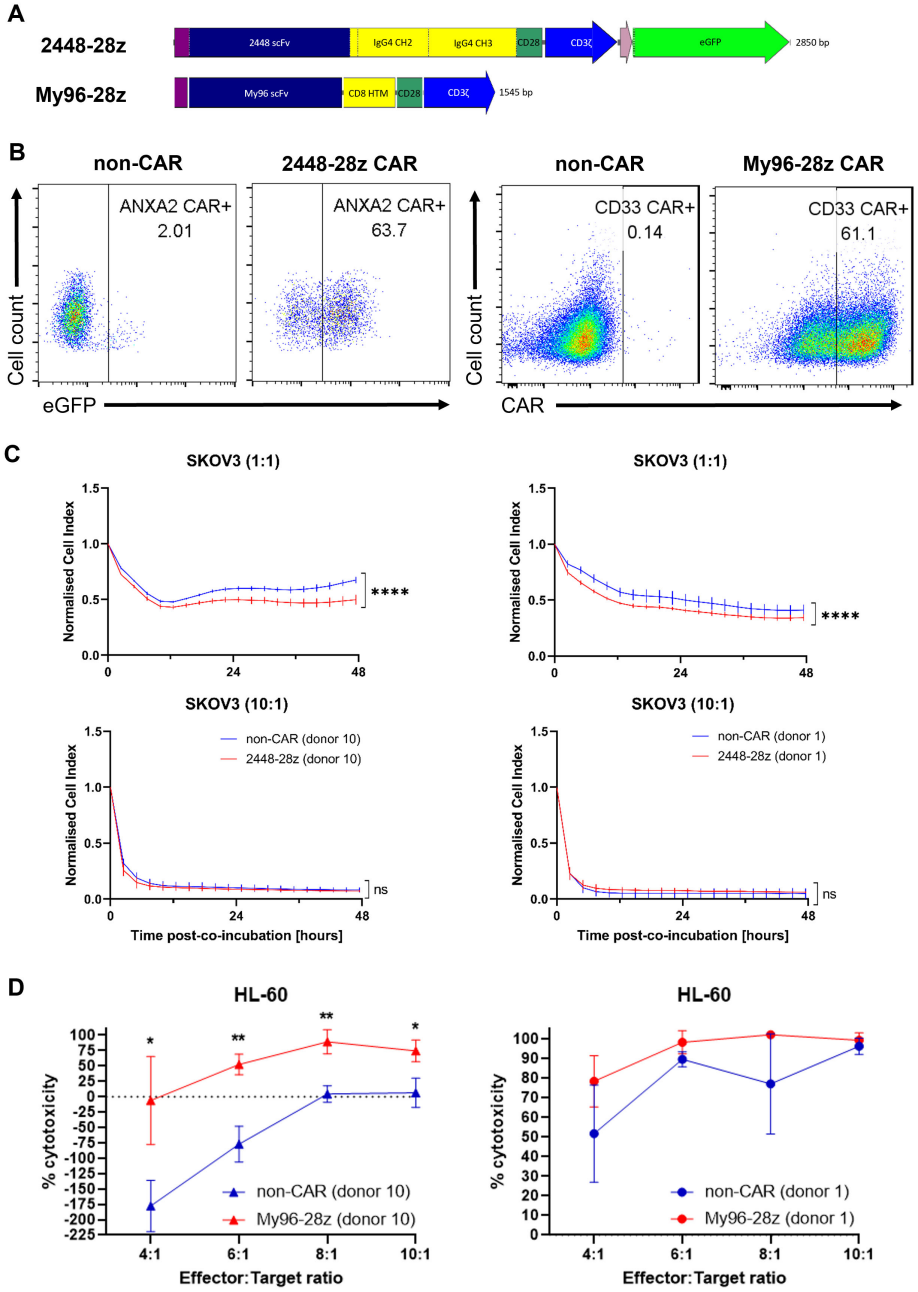
2448-28z and My96-28z CAR enhances anti-tumor cytotoxicity of NK cells. **(A)** Schematic diagram of 2448-28z and My96-28z CAR constructs cloned into pcDNA3.1(+) vector. **(B)** % eGFP as surrogate marker for 2448-28z CAR as assessed by flow cytometry (left) or % My96-28z CAR as assessed by Protein L staining followed by flow cytometry (right) in viable NK cells 24 h post-electroporation. **(C)** Cell index response curves of SKOV-3 co-cultured with 2448-28z CAR-NK cells in the xCELLigence system. **(D)** % cytotoxicity (calculated as described in Materials and Methods) of My96-28z NK cells against luciferase-expressing HL-60 cells 20 h following their co-incubation with NK cells derived from two different PBMC donors at the indicated E:T ratios. Data shown in **(C, D)** are the mean ± SD of technical triplicates of NK and tumor cell co-culture; multiple unpaired student’s t-tests, *, *p* < 0.05; **, *p* < 0.005; ****, *p* < 0.0001.

Following identification of the nucleofection program CM-137 for introducing CAR mRNA into NK cells, we further validated our optimized protocol using two, namely 2448-28z and My96-28z, CARs (**Figure 3A**). 2448-28z CAR bearing the single chain variable fragment (scFv) of an antibody (Ab) discovered by our lab targets the Annexin A2 antigen on tumor cells (15). Consistently, 2448-28z CAR-T cells killed annexin A2-expressing SKOV3 ovarian tumor cells to greater extent than non-CAR counterparts (16). My96-28z CAR was engineered using a publicly available scFv sequence based on an anti-CD33 Ab (17) and cloned into MSGV retroviral vector. When transduced into T cells, My96-28z CAR-T cells exhibited significantly enhanced cytotoxicity compared with mock-transduced non-CAR T cells against CD33-expressing tumor cell lines co-cultured for 48 h (**Supplementary Figure S2**). Thus, demonstration of proof-of-concept anti-tumor activity in T cells motivated their use in NK cells.

After 24 h post-electroporation of NK cells, the transfection efficiency of each CAR was verified against the non-CAR controls (**Figure 3B**) before *in vitro* co-cultures were initiated for the cytotoxicity assays. 2448-28z CAR-NK cells were observed to have better anti-tumor cytotoxic capacity compared to their non-CAR counterparts at E:T 1:1 and E:T 2:1 against SKOV3 ovarian cancer cells (**Figure 3C**, top panels and data not shown). However, there were no obvious differences at higher E:T ratios, presumably due to the overriding intrinsic cytotoxic functions of NK cells (**Figure 3C**, bottom panels). These observations were consistent across 4 different donors which we tested. Similarly, My96-28z NK cells derived from 2 different PBMC donors were more proficient than non-CAR NK cells at killing HL-60 acute myeloid leukemia (AML) cells (**Figure 3D**), although this was not evident in another AML cell line, MV4;11 (data not shown). Interestingly, the addition of NK (**Figure 3D**) or T cells (**Supplementary Figure S2**) to target tumor cells at lower E:T led to an initial expansion of tumor cells which has not been reported before. Although we are unable to explain this phenomenon observed in suspension but not adherent tumor cells, we observed that NK cells nonetheless killed tumor cells at higher E:T ratios although this differed greatly among NK cells derived from different PBMC donors. Of note, experimental results were obtained using different PBMC donors which demonstrated the robustness of our methodology.

### Optimized nucleofection condition is ideal for screening candidate CARs

**Figure 4:**
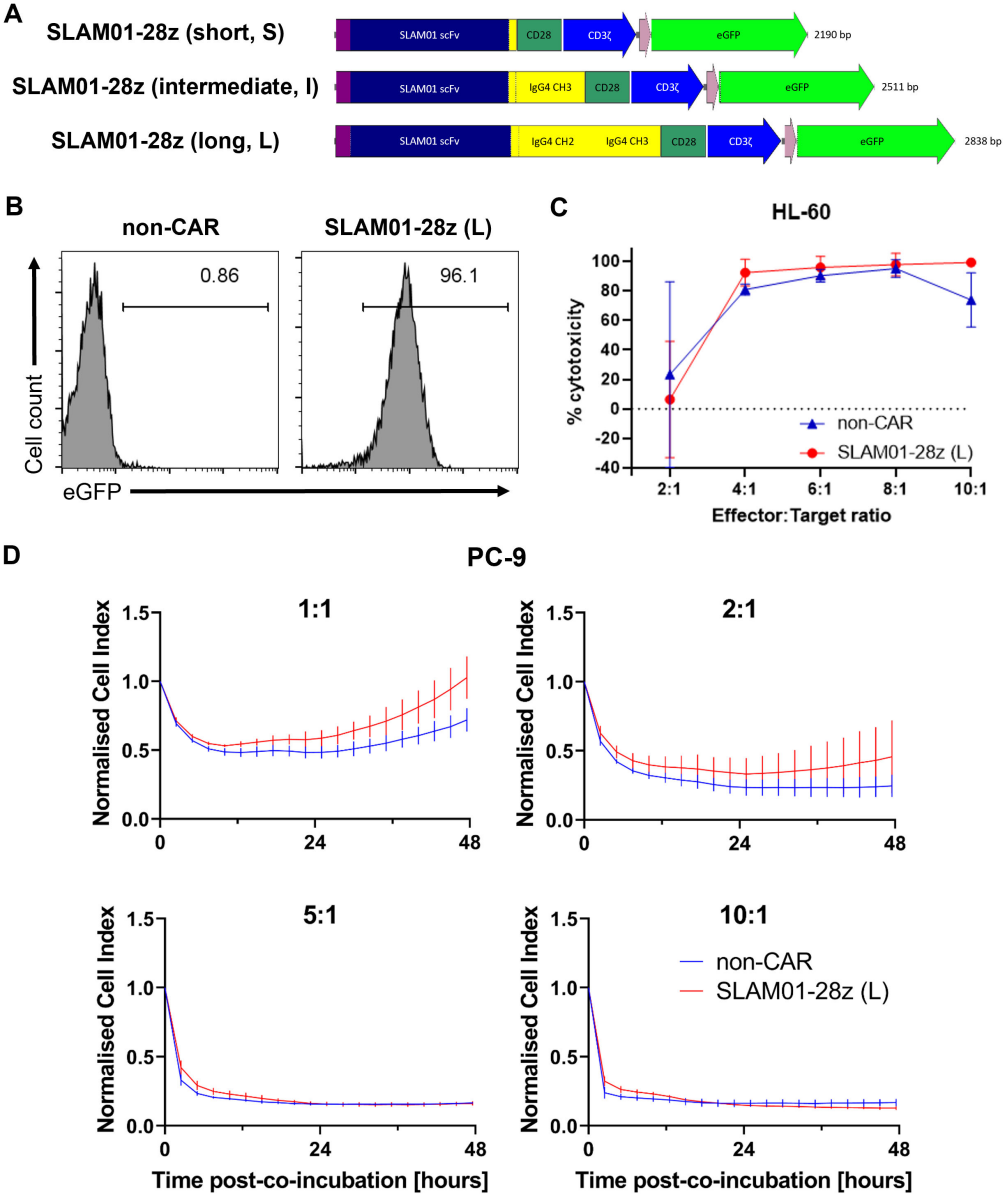
SLAM01-28z (long, L) CAR-NK cells exhibited anti-tumor cytotoxicity similar to that of non-CAR counterparts. **(A)** Schematic diagram of SLAM01-28z CAR constructs, with varying spacer lengths, cloned into pcDNA3.1(+) vector. **(B)** % eGFP^+^ in viable NK cells 24 h post-electroporation as assessed by flow cytometry. **(C)** % cytotoxicity (calculated as described in Materials and Methods) of SLAM01-28z (L) CAR-NK cells against luciferase-expressing HL-60 cells co-incubated at indicated E:T ratios for 20 (h). **(D)** Cell index response curves of PC-9 co-cultured with SLAM01-28z (L) CAR-NK cells in the xCELLigence system. Data shown in **(C, D)** are the mean ± SD of technical triplicates of NK and tumor cell co-culture; multiple unpaired student’s t-tests.

**Figure 5:**
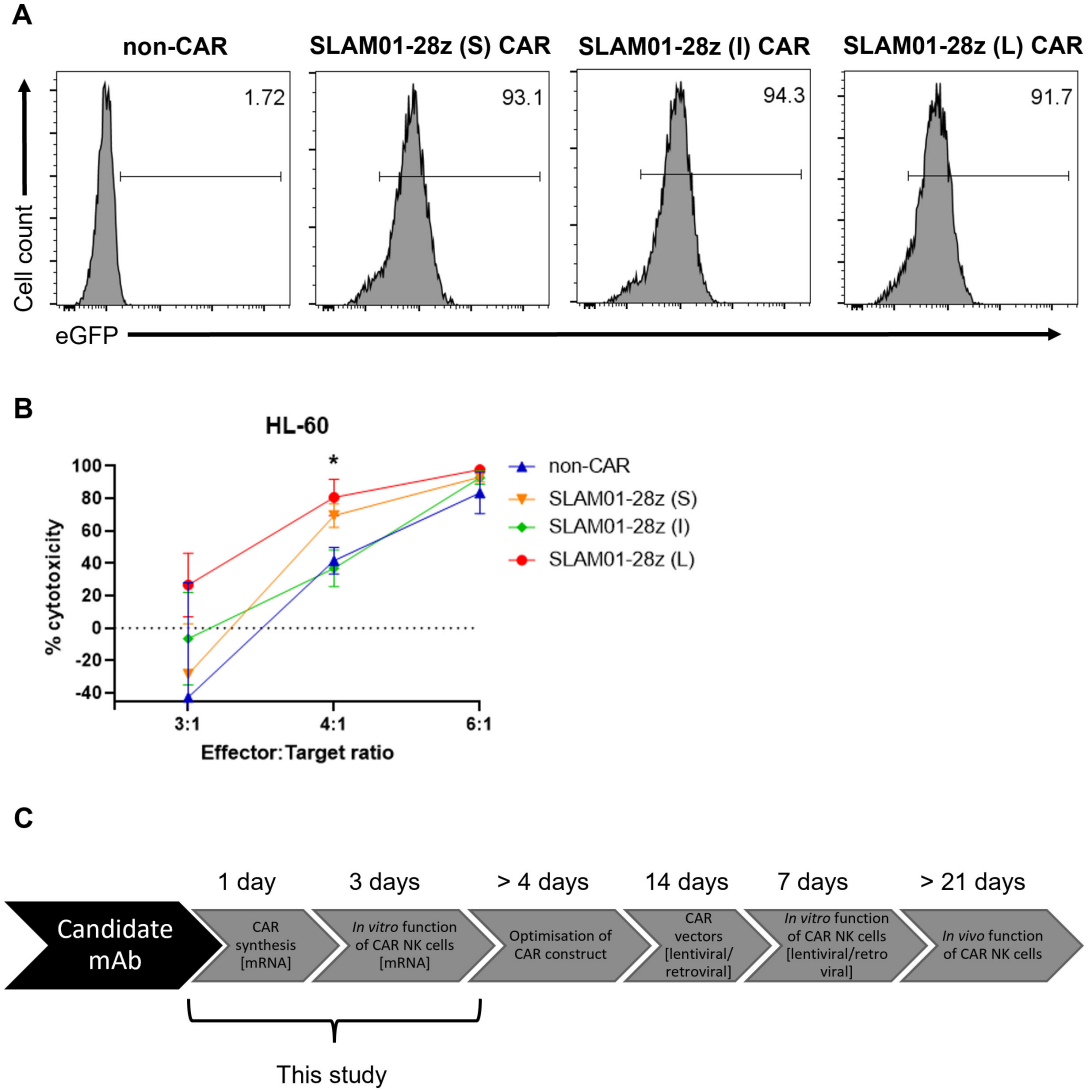
NK cells expressing SLAM01-28z CAR with varying spacer lengths have comparable anti-tumor cytotoxicity. **(A)** % eGFP^+^ in viable NK cells 24 h post-electroporation. **(B)** % cytotoxicity (calculated as described in Materials and Methods) of SLAM01-28z (S, I, L) CAR-NK cells against luciferase-expressing HL-60 cells co-incubated at indicated E:T ratios for 20 (h). **(C)** Schematic diagram showing our optimized workflow that can be adopted for future screening of CAR candidates in NK cells. Data shown in **(B)** are the mean ± SD of technical triplicates of NK and tumor cell co-culture; multiple unpaired student’s t-tests, *, *p* < 0.05.

We then proceeded to further assess three SLAM01-28z CAR candidates bearing short, intermediate and long spacer lengths for capacity to direct killing of tumor cells (**Figure 4A**). All three CARs recognize the Lewis X type glycan on SLAMF7 antigen expressed on target cells, e.g. HL-60 and PC-9 (**Supplementary Figure S3**). As before, we ensured that the CAR is adequately expressed in NK cells before carrying out the *in vitro* cytotoxicity assay (**Figure 4B**). This screening showed that SLAM01-28z (L) CAR-NK cells was not more effective than mock-transfected NK cells in killing both HL-60 (**Figure 4C**) and PC-9 (**Figure 4D**) as no significant difference in anti-tumor cytotoxicity was observed between SLAM01-28z (L) and non-CAR NK cells at all E:T ratios tested. Despite all three CARs being expressed at similar frequencies in NK cells (**Figure 5A**), NK cells carrying either S, I or L variant of SLAM01-28z CAR exhibited similar cytotoxicity against HL-60 cells compared with non-CAR NK cells (**Figure 5B**). Our proof-of-concept screening suggested that SLAM01-28z, though efficacious as an Ab-drug conjugate (data not shown) should not be pursued further as a CAR. Together, the results in **Figures 3**-**5** showed that NK cells transfected with different CARs exhibited different levels of cytotoxicity against target cells which are significantly or negligibly different from that of non-CAR counterparts. Hence, it is likely that the varying anti-tumor cytotoxicity levels observed are attributed to the CAR *per se* and not the nucleofection process, suggesting that our workflow of rapid nucleofection is a feasible strategy for transient CAR transgene expression in NK cells to prioritize best and deprioritize worst performing CAR candidates.

## Discussion

### NK cells with high fitness are successfully and reproducibly generated with our protocol

As a first step toward applying NK cells for immunotherapy, we derived a workflow that successfully and reproducibly generates NK cells with high numbers and high viability (**Figure 1**). Achieving viable cells is an important part of successful electroporation/transfection, as we have observed on rare occasions that NK cells having viability of lower than 50% do not express high levels of CAR (data not shown). Being able to generate high numbers of NK cells is also crucial for translation of our transfection workflow to clinical applications.

In our approach, we used irradiated and engineered K562 feeder cells for NK cell expansion. While non-feeder-based approaches have been tested, using feeder cells resulted in more robust and cost-effective expansion in our hands. Although the use of K562 chronic myeloid leukemia cells may pose a safety risk, the cells are irradiated at a high dosage of 100 Gy which sufficiently mitigates this risk. Moreover, final clinical NK products will be assessed for their critical quality attributes (CQAs) including being free of contamination from feeder cells prior to infusion.

### An optimized protocol for mRNA nucleofection into human NK cells can be used for screening CAR candidates

As Lonza’s nucleofector has not been reported to be used for CAR mRNA transfection into NK cells prior to our work, we embarked on this study to determine the most optimal program to be used for this purpose. In this study, we concluded that CM-137 is the most ideal program to be used to transfect NK cells, and that varying cell density between 0.5-1 × 10^6^ NK cells in 20 μl nucleocuvettes do not significantly alter the transfection efficiency (**Figure 2**). Subsequently, the same conditions encompassing the nucleofection program can be implemented on the Lonza platform for the purpose of clinical translation.

We applied the optimized program to test CARs containing three distinct scFv (**Figures 3A**, **4A**). The first two 2448-28z and My96-28z CARs we tested validated the robustness of our optimized protocol. Consistent with our previous work showing T cells harboring these CARs specifically lysed CAR antigen-expressing tumor cells, NK cells bearing the same CARs killed their respective target cells more efficiently than their non-CAR counterparts (**Figure 3**).

In this paper, we also described a phenomenon which, to our knowledge, has not been documented in the literature. The co-cultures of immune (NK or T) cells with suspension tumor cells consistently resulted in stimulation of expansion of the tumor cells. The killing effect by the immune cells overcome the stimulatory effects at higher E:T ratios. To provide a definitive mechanistic explanation of this observation is beyond the scope of this paper, but we propose that investigation of the molecular mechanism underlying the stimulatory versus cytotoxic effects of immune cells against suspension such as HL-60 AML tumor cells will be useful for further cytotoxicity studies of immune cells.

When we examined CAR candidates of interest, SLAM01-28z CAR variants bearing different spacer lengths directed similar tumor killing efficacy in NK cells (**Figures 4**, **5**). This is contrary to previous observations that SLAM01 functioned effectively as an Ab-drug conjugate. Nevertheless, we assert that our strategy is useful to continue screening CAR candidates for further pursuit toward clinical translation. The development time for optimizing CAR constructs is now 4 days with mRNA nucleofection instead of 2 weeks with retroviral transduction (**Figure 5C**). Upon identification of a potential CAR, we will adapt the CAR into a lentiviral or retroviral backbone which will be used for stable transduction of CAR into NK cells for further verification studies, such as preclinical *in vivo* assays. We have successfully utilized such a workflow for CAR-T cells (16), and aim to demonstrate that this is applicable to CAR-NK cells as the subject of future investigation.

## Data Availability

The raw data supporting the conclusions of this article will be made available by the authors, without undue reservation.
