# Supplementary material for: Nucleofection-based screening of chimeric antigen receptor candidates in human natural killer cells
Source: Front Immunol. 2025 Apr 3;16:1557766. doi: 10.3389/fimmu.2025.1557766 (PMC12003363; doi:10.3389/fimmu.2025.1557766)
Supplement: Supplementary file 1 [file Table1.docx]

Supplementary Material

# Supplementary Figures


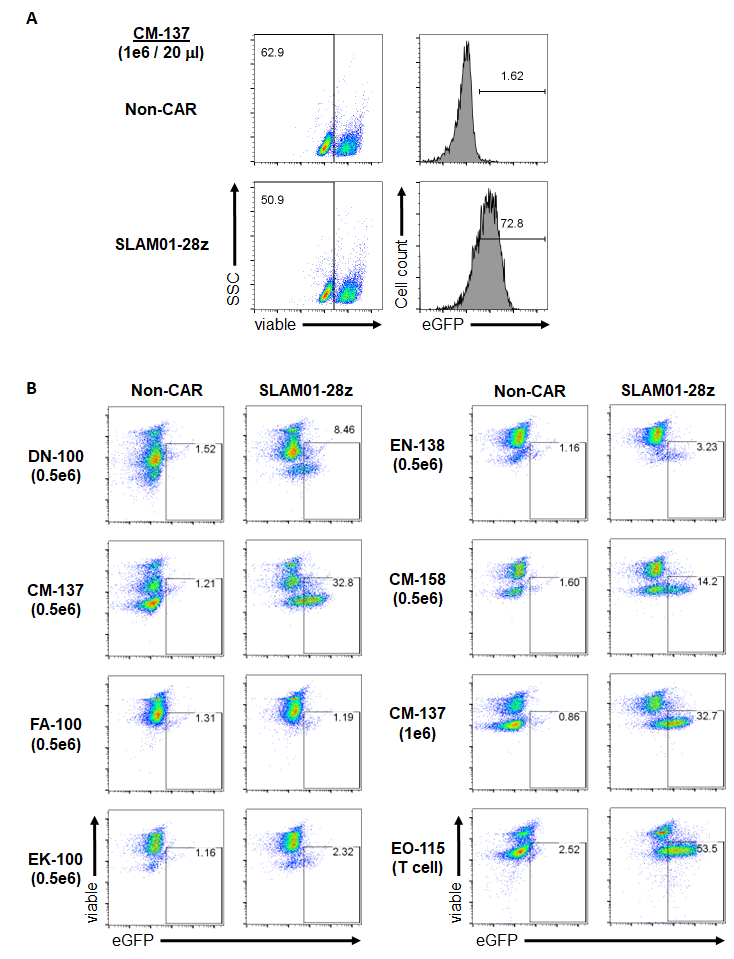


**Supplementary Figure 1.** CM-137 is an optimized program for nucleofection of CAR mRNA into NK cells. (A) Flow cytometry plots showing viability and eGFP expression of 1 × 10^6^ NK cells post-

24 h nucleofection in 20 µl nucleocuvettes using Lonza program CM-137. (B) Plots as in (A) of NK cells nucleofected with various nucleofection programs. T cells were used as electroporation control.


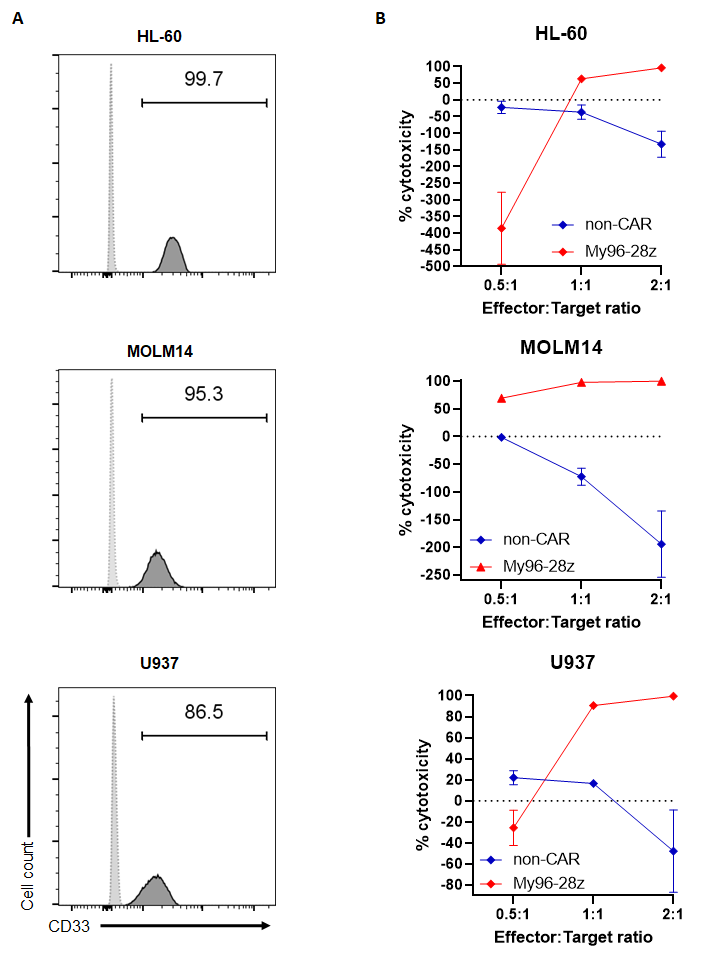


**Supplementary Figure 2.** My96-28z T cells show enhanced anti-tumor cytotoxicity. (A) Histograms showing CD33 expression (solid line, dark grey) across different cell lines. Unstained cells were used for negative gating (dotted line, light grey). (B) Percentage (%) cytotoxicity (calculated as described in Materials and Methods) of My96-28z T cells against 3 luciferase-expressing cell lines 20 h following their co-incubation with T cells at indicated E:T ratios.


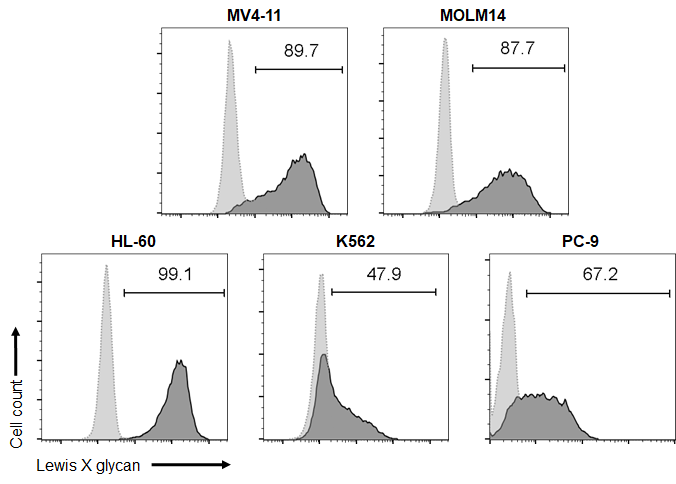


**Supplementary Figure 3.** Cell lines express varying intensities of target Lewis X glycan on SLAMF7 antigen. Histograms showing antigen expression (solid line, dark grey) across different cell lines. Unstained cells were used for negative gating (dotted line, light grey).

# Supplementary materials and method

**Flow cytometry**

Prior antibody staining, all cells were treated with Human TruStain FcX (Fc receptor blocking solution; BioLegend #422302). Expression of CD33 and Lewis X glycan on SLAMF7 antigen on target cell lines were assessed by CD33-BV421 (BD Biosciences #562854) and in-house generated antibody, respectively. 4’,6-diamidino-2-phenylindole dihydrochloride (DAPI; Biolegend #422801) solution or Hoechst 33342 (Invitrogen #H3570) solution were used to exclude dead cells. Samples (at least 20, 000 events) were acquired on MACSQuant X (Miltenyi Biotech) and data was analyzed with FlowJo software (TreeStar).

**Retroviral particle production and generation of transduced CAR-T cells**

Phoenix-GP cells stably expressing MoMLV gag-pol (ATCC #CRL-3215) were co-transfected with MSGV-based CAR constructs and pCMV-VSV-G (Addgene #8454) using FuGENE 6 (Promega). Transient retroviral supernatant was collected 48 h after transfection, filtered with 0.45 μm filter and used to transduce PG13 cells (ATCC #CRL-10686) by spinoculation at 600 × *g* for 2 h to generate cells which produce GaLV-pseudotyped CAR retrovirus (CAR PG13 cells) and cryopreserved. One week before activation of PBMCs, CAR PG13 cells were thawed and cultured to generate viral supernatant which was filtered, added and centrifuged at 1500 × *g* for 2 h to bind RetroNectin (rFN-CH-296; Takara Bio Inc. #T100B) pre-coated at 5.26 μg/cm^2^ in non-tissue culture-treated 6-well plates. PBMCs were activated in the presence of 50 ng/ml soluble anti-CD3 (OKT3; eBioScience) and 100 ng/ml anti-CD28 (CD28.2; eBioScience) antibodies and 20 IU/ml recombinant human IL-2 (Peprotech) for 2 days and activated T cells were transduced with CAR or not by respectively applying them to CAR or mock virus-bound RetroNectin at 600 × *g* for 30 min. Transduced T cells were expanded in the presence of 100 IU/ml IL-2 for further 3 days before coculture for luciferase-based cytotoxicity assay.
